# Coinfection of Cotton Plants with Watermelon Mosaic Virus and a Novel Polerovirus in China

**DOI:** 10.3390/v13112210

**Published:** 2021-11-03

**Authors:** Xiuling Yang, Min Du, Shupeng Li, Xueping Zhou

**Affiliations:** 1 State Key Laboratory for Biology of Plant Diseases and Insect Pests, Institute of Plant Protection, Chinese Academy of Agricultural Sciences, Beijing 100193, China; xlyang@ippcaas.cn (X.Y.); dumincaas@163.com (M.D.); shupengli123@163.com (S.L.); 2 State Key Laboratory of Rice Biology, Institute of Biotechnology, Zhejiang University, Hangzhou 310058, China

**Keywords:** mixed infection, cotton leaf roll virus, watermelon mosaic virus, polerovirus, potyvirus

## Abstract

Cotton is the most important fiber crop worldwide. To determine the presence of viruses in cotton plants showing leaf roll and vein yellowing symptoms in Henan Province of China, a small RNA-based deep sequencing approach was performed. Analysis of the de novo-assembled contigs followed by reverse transcription PCR allowed the reconstruction of watermelon mosaic virus and an unknown virus. The genome of the unknown virus was determined to be 5870 nucleotides in length, and has a genomic organization with characteristic features of previously reported poleroviruses. Sequence analysis revealed that the virus was closely related to, but significantly different from, cotton leafroll dwarf virus, a polerovirus of the family *Solemoviridae*. This virus had less than 90% amino acid sequence identity in the products of both ORF0 and ORF1. According to the polerovirus species demarcation criteria set by the International Committee on Taxonomy of Viruses, this virus should be assigned to a new polerovirus species, for which we propose the name “cotton leaf roll virus”.

## 1. Introduction

Plant viruses have caused significant yield and quality losses to a wide range of economically important crops [1]. As obligate intracellular parasites, plant viruses are impossible to be cured with antiviral agents, making plant viral diseases very difficult to control in field. Early diagnosis and precise identification of viral diseases are therefore crucial for controlling the pandemic of plant viruses. In recent years, the development of deep sequencing-based discovery of viruses allows us to rapidly identify accurate virus species without prior knowledge of the viral sequences [2,3,4,5,6].

The *Potyviridae* is the largest family of plant RNA viruses that are ascribed to substantial yield losses to agricultural, pastoral, horticultural, and ornamental crops globally [7]. Twelve genera have been classified based on the host range, genomic features, and phylogeny of *Potyviridae* member viruses. Viruses belonging to the genus *Potyvirus* constitute the largest group of the family *Potyviridae* and are transmitted by aphids. The typical genome of potyviruses has a single-stranded, linear, positive RNA that is made up of approximately 10,000 nucleotides. It encodes a long open reading frame (ORF) that is processed into functional proteins by three self-encoded proteases. An additional small ORF, PIPO, is formed from RNA polymerase slippage during viral genome replication [7]. Besides its own importance in causing damages to crops, mixed infections are frequently observed under natural conditions between viruses from the *Potyvirus* genus and viruses from other genera. Although the outcomes of coinfection with two individual viruses may vary, coinfection of a potyvirus with another distinct virus often results in an enhancement of symptom severity and yield loss [8]. For example, a combination infection of maize plants with maize chlorotic mottle virus and sugarcane mosaic virus causes maize lethal necrosis in many regions of the world, while a single infection of maize plants with each virus causes milder symptoms [9,10].

The *Solemoviridae* is another important family of plant linear (+) ssRNA viruses that are divided into four genera (*Enamovirus*, *Polemovirus*, *Polerovirus,* and *Sobemovirus*) (https://talk.ictvonline.org/taxonomy/, accessed on 20 August 2021) [11]. Viruses within the *Polerovirus* genus are transmitted by aphids and possess a genome of about 5300 to 6000 nucleotides in length. The typical genome of a polerovirus encodes seven ORFs (ORF0, ORF1, ORF1-ORF2, ORF3, ORF3a, ORF4, and ORF3-ORF5) [12]. The ORF0 encodes the P0 protein that acts as a suppressor of RNA silencing. ORF1 and ORF1-ORF2, which overlap with one another, encode the P1 and P1-P2 proteins involved in virus replication. ORF3 encodes the coat protein P3, and ORF4 is thought to encode the movement of protein P4. Translation via read-through of the P3 stop codon generates the P3-P5 protein that is related to aphid transmission and virus accumulation in plants. P3a, the product of a recently described small ORF3a, is required for virus long-distance movement in plants [12].

Cotton is the most important fiber crop worldwide. It is also an excellent multipurpose crop providing a source of feed, foodstuff, biofuel, and oil production. Previous studies have shown that, in some of the major cotton-producing countries, cotton crops are often severely affected by diseases caused by infection with cotton leaf curl begomoviruses or and cotton leafroll dwarf virus [13,14,15]. In this study, cotton plants showing typical symptoms of plant virus infection were collected from the Henan province of China. A coinfection of watermelon mosaic virus and a novel member of the genus *Polerovirus* were identified from the diseased cotton plants through Illumina-based deep sequencing.

## 2. Materials and Methods

### 2.1. Sample Collection and RNA Extraction

**Figure 1:**
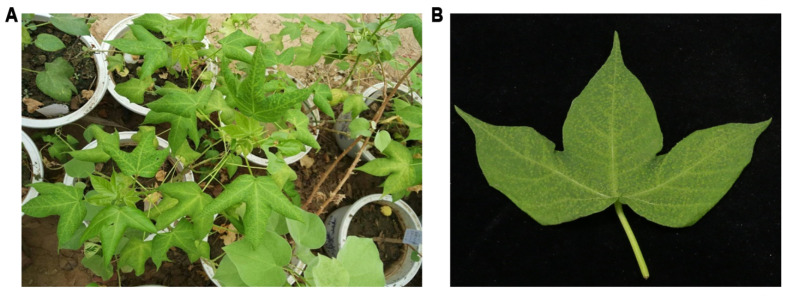
Symptoms on virus-infected cotton plants collected from the Henan province of China. (**A**) Symptoms on whole cotton plants. (**B**) A close view of the symptoms of diseased cotton leaf.

Cotton plants showing leaf roll and vein yellowing symptoms were collected from Anyang City, in the Henan province of China, in May 2016 (Figure 1). Leaf tissues were immediately frozen in liquid nitrogen and stored at −80 °C until processing. Total RNA was extracted from diseased leaf tissues using Tiangen RNA extraction buffer following the manufacturer’s instructions (Tiangen, Beijing, China).

### 2.2. Small RNA Library Construction, Sequencing, and Data Processing

Small RNA library was constructed as described [16]. In brief, low-molecular-weight RNAs were enriched using the Small RNA Sample Pre Kit (Illumina, San Diego, CA, USA) and sequentially ligated a 3′ adapter and a 5′ adapter. The final purified ligation products were reverse transcribed into cDNA using SuperScript^TM^ III reverse transcriptase (Invitrogen, Carlsbad, CA, USA). The first-strand cDNA was then PCR amplified and subjected to Illumina Hiseq2000 sequencing (Novogene, Beijing, China). The raw reads were filtered by trimming adapter sequences and removal of poly A/T/C/G reads and low-quality reads. The clean sRNA reads ranging from 18 to 26 nts in length were mapped to the cotton genome, and the clean unmapped sRNA reads were assembled using the Velvet program [17]. Assembled contigs were compared against the Genbank database using BLASTN (http://www.ncbi.nlm.nih.gov/, accessed on 15 January 2017).

### 2.3. RT-PCR Validation, Full-Length Genome Amplification, and Sequencing

The presence of candidate viruses in diseased leaf tissues was validated by reverse transcription PCR (RT-PCR). Primers were designed according to the sequences of assembled contigs of virus-derived small interfering RNAs (vsiRNAs).

Total RNA was isolated from cotton leaf tissues using Tiangen RNA extraction buffer (Tiangen). For cloning of the full-length viral genomes, first-strand cDNA synthesis was performed using the SuperScript^TM^ II First-Strand Synthesis System (Invitrogen) with a random hexamer and virus-specific primers (Table S1). Amplification of the different fragments of candidate viruses was carried out using TransStart FastPfu DNA polymerase (TransGen Biotech, Beijing, China). The resulting PCR products were purified and cloned into a pEASY-Blunt simple vector (TransGen). The recombined clones were sequenced with universal primer pairs M13F/M13R and walking primers via Sanger sequencing (TsingKe Biotech Co., Beijing, China). The 5′-terminal and 3′-terminal sequences of candidate viruses were obtained through 5′ RACE and 3′ RACE systems using a SMARTer RACE cDNA Amplification Kit following the manufacturer’s protocol (Clontech, Mountain View, CA, USA). At least three independent clones were sequenced for each fragment.

For detection of the negative strand of watermelon mosaic virus, reverse transcription was performed using a tagged primer WMV-NS-RT (Table S1, tag in lowercase, WMV nt 7375–7396 in uppercase). PCR was conducted with primers for the tag (WMV-NS-tag) and WMV (WMV-NS-R, complementary to WMV nt 8119–8140). For detection of the positive strand of WMV, the primer WMV-NS-R was used in reverse transcription, and the primer pair WMV-4F/WMV-NS-R was used for PCR amplification.

### 2.4. Viral Genome Characterization

Sequences were processed using the Lasergene 7 SoftWare (Lynnon Biosoft, Quebec, QC, Canada). Open reading frames (ORFs) were predicted using the ORF Finder program (https://www.ncbi.nlm.nih.gov/orffinder/, accessed on 15 March 2017), and verified by comparison with previously annotated ORFs from CLRDV and WMV reference sequences deposited in GenBank.

### 2.5. Phylogenetic Analyses

Sequences were aligned using MUSCLE, implemented with the MEGA software version 6.06. Phylogenetic trees were constructed with MEGA6 software via the neighbor-joining method with 1000 bootstrap replicates [18]. Pairwise sequence comparisons were performed using the Sequence Demarcation Tool (SDT) v1.2 [19].

### 2.6. Quantitative RT-PCR of Viral RNA

Quantitiave RT-PCR (qRT-PCR) was performed using a TransStart Green qPCR Supermix (Transgene, Beijing, China) and the Roche LightCycler 96 system (Roche, Germany) following the manufacturer’s recommendations. The relative expression of viral RNA was determined by normalizing to the expression of the internal control *UBQ7* using the comparative Ct method (2^−ΔΔCt^).

## 3. Results

### 3.1. Identification of Two RNA Viruses in Cotton Plants Using Next-Generation Sequencing

To identify virus(es) present in the diseased cotton plants (Figure 1), leaf tissues were collected and subjected to deep sequencing using the Illumina Hiseq2000 platform. A total of 13,608,058 clean reads were obtained after filtering the 14,246,025 raw reads by trimming adapter sequences, poly A/T/C/G reads, and low-quality reads. After mapping siRNAs of 18–26 nts in size to the cotton genome, the unmapped 1,746,854 sRNA reads were de novo assembled into contigs using the Velvet program. Comparison of the assembled contigs to the GenBank database showed that 47 contigs covering 5995 nts were mapped to watermelon mosaic virus (WMV) (genus *Potyvirus*, family *Potyviridae*), and 28 contigs covering 3326 nts were annotated to have a high similarity to the cotton leafroll dwarf virus (CLRDV) (genus *Polerovirus*, the family *Solemoviridae*).

To validate and obtain the full-length viral genome sequences, several RT-PCR amplifications were carried out using primers (Table S1) designed based on the contig sequences. The 5′-terminus and 3′-terminus of viral genomes were subsequently amplified using 5′ RACE and 3′ RACE systems. Cloning, sequencing, and assembly of the different fragments produced two full-length genomes of 10,031 nts and 5870 nts in length, respectively. BLASTN search using the assembled 10,031-nt and 5870-nt viral genomes showed that the 10,031-nt genome shared the highest nucleotide sequence identity with WMV strain WMV-Li (MG194418.1), with a nucleotide identity of 96.5%, while the 5870-nt genome viral sequences shared the highest nucleotide identity with CLRDV isolate M5 atypical (91.74% identity; Table 1). The results suggested that one virus is an isolate of WMV and the other is a polerovirus.

### 3.2. Characterization of the WMV Isolate

**Figure 2:**
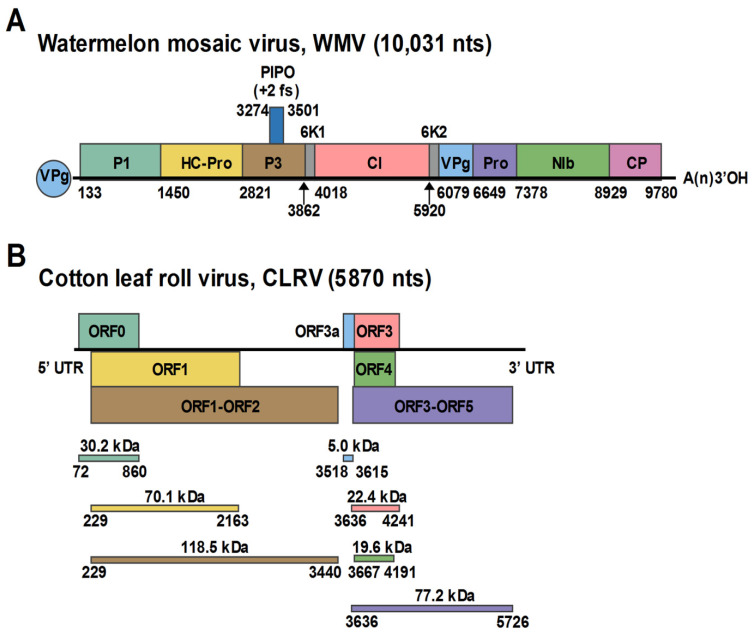
Schematic representation of the genome organization of watermelon mosaic virus (**A**) and cotton leafroll virus (**B**). Open reading frames (ORFs) and deduced products of each RNA are shown as indicated as colored boxes.

**Figure 3:**
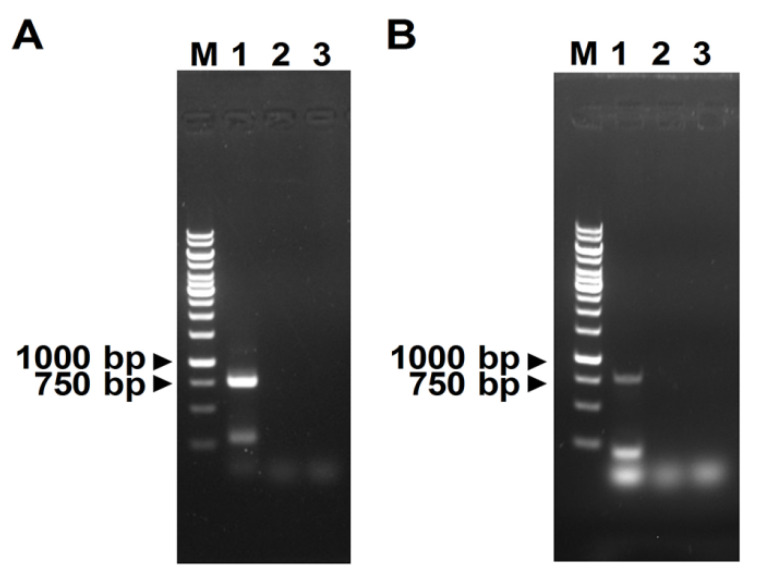
Strand-specific RT-PCR detection of the positive-strand (**A**) and negative-strand (**B**) RNA of watermelon mosaic virus. Lane M, DNA marker (Thermo). Lane 1 and Lane 2 indicate cDNA transcribed from diseased and healthy cotton leaves, respectively. Lane 3 indicates that double-distilled water was used as the negative control.

Similar to members in the genus *Potyvirus*, the Henan isolate of WMV encoded a single polyprotein (133–9780 nt) which was predicted to contain 11 protein products, including 10 mature products proteolytically processed by viral proteases and an 11th protein P3N-PIPO embedded in P3 by +2 ribosomal frameshifting (Figure 2A). A phylogenetic tree generated using the complete sequences of the Henan isolate and other available WMV sequences from the GenBank database showed that the Henan isolate is most closely related to the WMV strain WMV-Li. Pairwise identity analysis using the polyprotein sequences showed that the Henan isolate shared 96.5% nucleotide identity and 98.5% amino acid identity to the WMV strain WMV-Li. Using strand-specific RT-PCR, the positive-strand WMV RNA was detected in the diseased cotton leaf tissues (Figure 3A). Furthermore, the negative-strand WMV RNA, which can only be produced during viral replication, could also be detected in diseased tissues, indicating the replication of WMV in the collected cotton leaves (Figure 3B). According to the species demarcation criteria of the family *Potyviridae*, the Henan isolate is an isolate of WMV, with proposed designation WMV-[Henan] (GenBank Accession Number: OK050524).

### 3.3. Characterization of the Polerovirus

Similar to previously reported poleroviruses, the genomic organization of the Henan isolate comprised 5′ block and 3′ block that are separated by a 79-nt noncoding sequences. The 5′ block consisted of three predicted ORFs: ORF0, ORF1, and ORF1-ORF2. The 3′ block contained four ORFs, ORF3, ORF4, ORF3-ORF5, and an additional short non-AUG initiated ORF, called ORF3a, that is positioned upstream of ORF3 (Figure 2B). The 5′ UTR included 71 nts that start with ACAAAA, a feature conserved in many poleroviruses, and the 3′ UTR contained 145 nts.

The closest relatives of the 5870-nt viral sequences are the nine CLRDV isolates previously identified from Argentina, Brazil, and USA [14,20,21,22,23]. All the nine available CLRDV complete genome sequences were retrieved from the GenBank Database, and pairwise sequence identity was analyzed using SDT1.2. As shown in Table 1, the Henan isolate shares 90.92% to 91.74% nucleotide identity with the previously reported nine isolates of CLRDV (Table 1). Pairwise sequence identity comparison of the seven individual ORFs of the Henan isolate to the nine CLRDV isolates indicated that the predicted ORF0, encoding the predicted 30.3-kDa P0 protein, was the most divergent, and shared 85.37% to 86.51% identity at the nucleotide level and 76.92% to 80.7% identity at the amino acid level (Table 1). Sequences located within the ORF1 coding region displayed the second most divergent variability, with 88.08% to 89.00% nucleotide identity and 84.56% to 86.43% amino acid identity (Table 1). The ORF1-ORF2, which encodes the predicted 118.7-kDa viral RNA-dependent RNA polymerase, shared 90.33–91.08% and 89.61–90.82% identity at the nucleotide and amino acid levels, respectively (Table 1). The ORF4, which is predicted to encode a 19.6-kDa movement protein, shared a 94.10–95.05% nucleotide identity and 86.78–90.23% amino acid identity, respectively (Table 1). A high identity with less than 10% divergence was observed within the coding regions of ORF3, ORF3a, and ORF3-ORF5 (Table 1).

**Figure 4:**
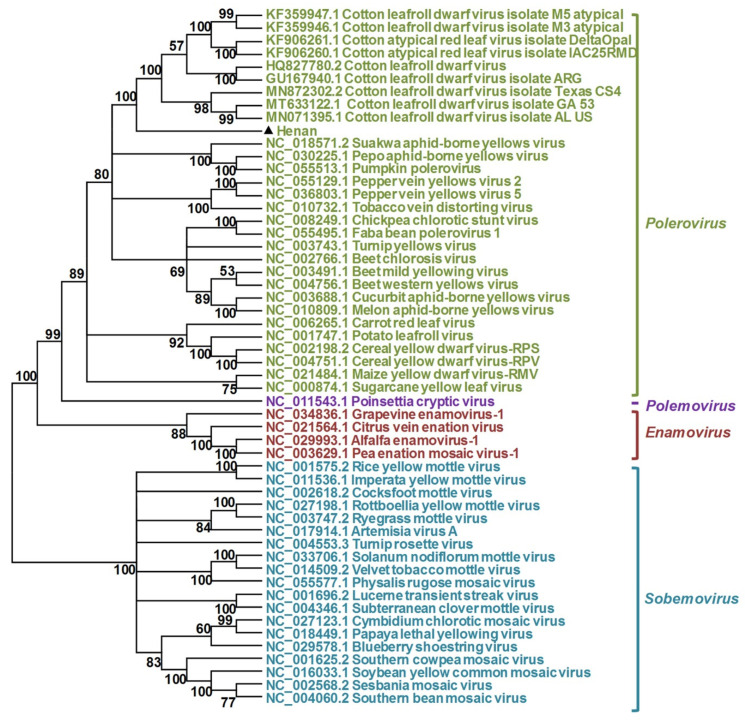
Phylogenetic analysis of cotton leaf roll virus and selected member viruses of the family *Solemoviridae* based on complete genome sequences. The accession number of each selected solemovirus is shown as indicated. The phylogenetic tree was constructed using the neighbor-joining method implemented with MEGA6, using a bootstrap replicates of 1000. Numbers shown next to the branches represent bootstrap values from 1000 replicates.

**Figure 5:**
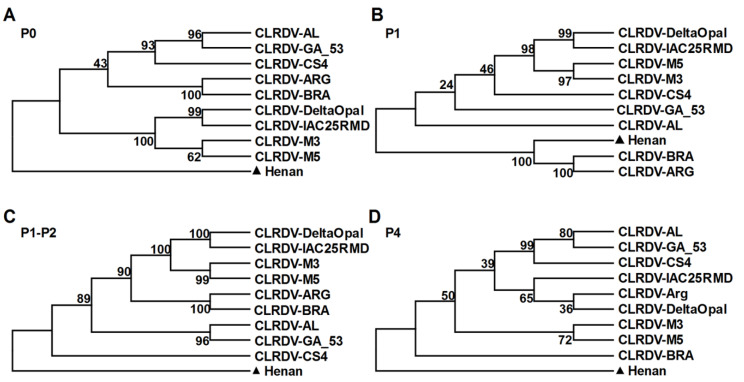
Phylogenetic analysis of cotton leaf roll virus and the nine previously reported cotton leafroll dwarf virus (CLRDV) isolates from Argentina, Brazil, and the United States using the amino acid sequences of the ORF0-encoded P0 protein (**A**), ORF1-encoded P1 (**B**), ORF1-ORF2-encoded P1-P2 protein (**C**), and ORF4-encoded P4 protein (**D**), respectively. The phylogenetic trees were generated using the neighbor-joining method implemented with MEGA6, using a bootstrap replicates of 1000. Numbers shown next to the branches correspond to bootstrap values from 1000 replicates.

To better understand the relationship of the Henan isolate to other poleroviruses, a phylogenetic tree was constructed based on the full-length genomic sequences of the Henan isolate and members of the family *Solemoviridae.* As shown in Figure 4, the Henan isolate grouped with viruses belonging to the genus *Polerovirus* and was most closely related to the nine CLRDV isolates. A closer view of the phylogenetic tree showed that the Henan isolate was in a distinct subclade from the nine CLRDV isolates (Figure 4). Phylogenetic trees generated using the amino acid sequences of ORF0, ORF1, ORF1-ORF2, and ORF4 indicated that the gene products of ORF0, ORF1-ORF2, and ORF4 (P0, P1-P2, P4) were distinct from the two genotypes of CLRDV, and the gene product of ORF1 (P1) formed a distinct clade with the CLRDV isolates collected from Argentina and Brazil (Figure 5). Currently, the accepted species demarcation criterion for the genus *Polerovirus* is based on the threshold of 10% difference in the amino acid sequence of any viral product. Taken into account the sequence divergence and the phylogenetic analysis, the Henan isolate is considered to represent a distinct species of the genus *Polerovirus*, for which the name cotton leaf roll virus (CLRV) is proposed (GenBank Accession Number: OK050525).

### 3.4. Relative Abundance of WMV and CLRV

**Figure 6:**
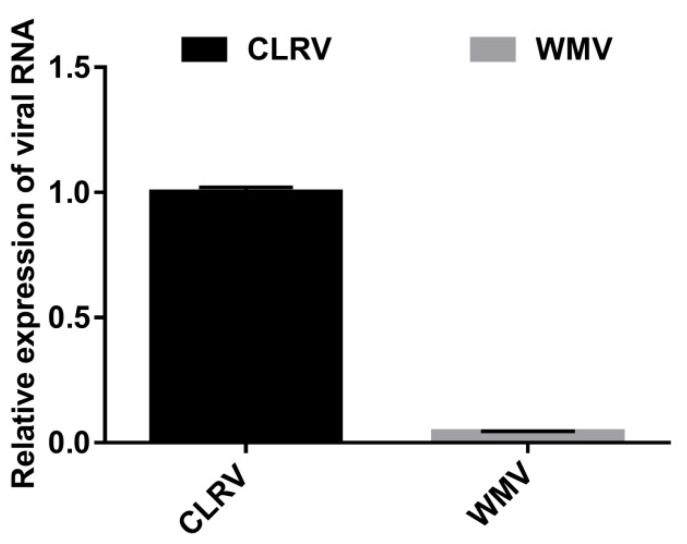
Quantitative RT-PCR analysis of the RNA accumulation of watermelon mosaic virus (WMV) and cotton leaf roll virus (CLRV). The expression of *UBQ7* was used as a reference control. The accumulation of CLRV and WMV RNA is represented relative to *UBQ7* using the comparative *Ct* method (2^−ΔΔCt^). The expression of CLRV was set as 1. Error bars represent the standard deviation of each data set.

To determine the abundance of WMV and CLRV in the collected cotton plants, qRT-PCR was used to analyze the relative expression of WMV and CLRV RNA. The results showed that the RNA accumulation level of CLRV was about 22 fold to that of WMV (Figure 6), suggesting that CLRV was far more abundant than WMV in the infected cotton leaves.

## 4. Discussion

The cutting-edge deep sequencing technology and bioinformatics have been proven to be a reliable and efficient approach for virus identification, which does not rely on prior knowledge or sequence of the virus of interest. Therefore, the development of this technology has boosted the discovery of novel viruses. In this study, we identified and characterized coinfection of cotton plants with a watermelon mosaic virus and a novel member of the *Polerovirus* genus.

The polerovirus identified in this study shares a typical genome structure with known poleroviruses. Phylogenetic analysis of the complete genome sequences to reported typical species of the family *Solemoviridae* also revealed that the identified virus grouped with poleroviruses, but separated from members of the *Enamovirus, Polemovirus*, and *Sobemovirus* genera. A detailed BLASTn and phylogenetic analysis showed that this virus was closely related to CLRDV, a polerovirus that has been described to cause cotton blue disease in Argentina, Africa, Asia, Brazil, and the United States [14,20,21,23,24,25]. The species demarcation criterion for the *Polerovirus* genus utilizes a >10% amino acid sequence difference in any of the viral products as a new species threshold [26]. In the case of CLRDV, it was proposed that, if the P0 coding region is the only ORF whose amino acid sequences exceed the 10% pairwise distance cutoff, the isolates represent different genotypes of a single viral species [22]. Sequence analysis of the polerovirus identified in this study showed that both the aa sequences of P0 and ORF1 shared <90% aa identity from the other known poleroviruses, suggesting that the Henan isolate obtained in this study is a novel polerovirus, for which cotton leaf roll virus is proposed.

Coinfections of the same host plant with heterologous viruses, such as potato virus Y (family *Potyviridae*, genus *Potyvirus*, PVY) and potato leafroll virus (family *Solemoviridae*, genus *Polerovirus*, PLRV) in potato cropping systems, are common in nature [27]. However, this is the first time a member of the *Potyvirus* has been found to co-infect with a polerovirus in cotton plants. The biological and epidemiological consequences of mixed viral infection are unpredictable and largely depend on the combinations of virus–virus and virus–host interactions, which are usually categorized as synergistic and antagonistic interactions [27]. Synergistic interactions of viruses during mixed infection can change disease development, virus concentration, tissue tropism, or host range. Previous studies of the effect of potyviruses on other virus accumulation have shown that potyviruses may facilitate multiple infections by additional viruses because HC-Pro, a potyvirus-specific RNA silencing suppressor, facilitates replication of other viruses in some cases [28]. While primarily phloem-limited and not mechanically transmissible, co-infection of *Nicotiana clevelandii* by PLRV and PVY increases PLRV accumulation and helps PLRV to invade non-phloem tissue [29]. An interesting parallel also exists in a previous study on mixed infection of PLRV and the umbravirus pea enation mosaic virus-2 (PEMV-2). In this synergism, PEMV-2 altered the transmission mode of PLRV and assists PLRV to invade mesophyll tissues and to be transmitted mechanically between plants [30]. Since both potyviruses and poleroviruses are transmitted by aphid, the mixed infection of WMV and CLRV in this study may be attributed to their transmission by aphids in the field. Although we could not compare virus titer between a single infection of each virus and the mixed infection of these two viruses in this study, qRT-PCR analysis of the relative abundance of WMV and CLRV RNA showed that the accumulation level of CLRV is much higher than that of WMV in the collected cotton samples. Future studies addressing the effect of WMV and CLRV interactions on disease development or on virus titer could indicate the biological and epidemiological consequences of this type of mixed infection.

## Figures and Tables

**Table 1:** Pairwise nucleotide and amino acid sequence identities of the viral open reading frames for the Henan isolate and isolates of cotton leafroll dwarf virus calculated using Sequence Demarcation Tool software v1.2.

Isolate	Nucleotide Identity (%) of Complete Genome	Nucleotide and Amino Acid Identity (%, nt/aa)						
		ORF0/P0	ORF1/P1	ORF1-ORF2/P1-P2	ORF3/P3	ORF3a/P3a	ORF4/P4	ORF3-ORF5/P3-P5
CLRDV-AL	90.92	85.86/80.00	88.08/85.02	90.33/90.17	93.4/96.02	98.55/100	94.10/86.78	92.29/92.80
CLRDV-ARG	91.46	85.75/80.77	88.54/85.18	90.83/90.45	93.56/95.02	99.28/100	94.28/87.93	92.91/93.66
CLRDV-CS4	91.33	85.6/78.85	88.96/86.43	90.92/90.64	93.73/96.02	98.55/100	94.48/87.93	92.48/93.66
CLRDV-Delta	91.55	86.00/76.92	88.28/84.56	90.80/89.70	93.73/96.02	99.28/100	94.67/89.08	93.06/94.38
CLRDV-GA53	91.45	85.37/79.23	88.65/85.34	91.02/90.26	93.40/96.02	99.28/100	94.1/87.36	92.43/93.08
CLRDV-HQ	91.7	86.13/80.77	89.00/85.80	91.08/90.82	94.22/96.02	97.82/100	95.05/90.23	93.20/94.38
CLRDV-IAC	91.48	86.13/77.31	88.34/84.71	90.77/89.89	93.73/96.02	99.28/100	94.67/89.08	92.86/94.38
CLRDV-M5	91.74	86.26/78.46	88.75/85.02	91.05/89.79	94.06/96.02	98.55/100	94.67/89.08	93.10/93.80
CLRDV-M3	91.7	86.51/79.23	88.75/85.02	91.01/89.61	92.73/96.52	98.55/100	94.28/87.93	93.15/94.24

## Supplementary Materials

The following is available online at https://www.mdpi.com/article/10.3390/v13112210/s1, Table S1: Sequences of primers used for amplification of cotton leaf roll virus (CLRV) and watermelon mosaic virus (WMV).

## References

[1] Nicaise V. (2014). Crop immunity against viruses: Outcomes and future challenges. Front. Plant Sci..

[2] Wu Q., Luo Y., Lu R., Lau N., Lai E.C., Li W.X., Ding S.W. (2010). Virus discovery by deep sequencing and assembly of virus-derived small silencing RNAs. Proc. Natl. Acad. Sci. USA.

[3] Chen S., Jiang G., Wu J., Liu Y., Qian Y., Zhou X. (2016). Characterization of a novel polerovirus infecting maize in China. Viruses.

[4] Xuan Z., Xie J., Yu H., Zhang S., Li R., Cao M. (2021). Mulberry (*Morus alba*) is a new natural host of citrus leaf blotch virus in China. Plant Dis..

[5] Ma Y., Navarro B., Zhang Z., Lu M., Zhou X., Chi S., Di Serio F., Li S. (2015). Identification and molecular characterization of a novel monopartite geminivirus associated with mulberry mosaic dwarf disease. J. Gen. Virol..

[6] Fu S., Zhang T., He M., Sun B., Zhou X., Wu J. (2021). Molecular characterization of a novel wheat-infecting virus of the family *Betaflexiviridae*. Arch. Virol..

[7] Yang X., Li Y., Wang A. (2021). Research advances in potyviruses: From the laboratory bench to the field. Annu. Rev. Phytopathol..

[8] Revers F., Antonio Garcia J. (2015). Molecular biology of potyviruses. Adv. Virus Res..

[9] Redinbaugh M.G., Stewart L.R. (2018). Maize lethal necrosis: An emerging, synergistic viral disease. Annu. Rev. Virol..

[10] Wang Q., Zhang C., Wang C., Qian Y., Li Z., Hong J., Zhou X. (2017). Further characterization of maize chlorotic mottle virus and its synergistic interaction with sugarcane mosaic virus in maize. Sci. Rep..

[11] Walker P.J., Siddell S.G., Lefkowitz E.J., Mushegian A.R., Adriaenssens E.M., Alfenas-Zerbini P., Davison A.J., Dempsey D.M., Dutilh B.E., Garcia M.L. (2021). Changes to virus taxonomy and to the International Code of Virus Classification and Nomenclature ratified by the International Committee on Taxonomy of Viruses (2021). Arch. Virol..

[12] Smirnova E., Firth A.E., Miller W.A., Scheidecker D., Brault V., Reinbold C., Rakotondrafara A.M., Chung B.Y., Ziegler-Graff V. (2015). Discovery of a small non-aug-initiated ORF in poleroviruses and luteoviruses that is required for long-distance movement. PLoS Pathog..

[13] Sattar M.N., Kvarnheden A., Saeed M., Briddon R.W. (2013). Cotton leaf curl disease—An emerging threat to cotton production worldwide. J. Gen. Virol..

[14] Agrofoglio Y.C., Delfosse V.C., Casse M.F., Hopp H.E., Kresic I.B., Distefano A.J. (2017). Identification of a new cotton disease caused by an atypical cotton leafroll dwarf virus in Argentina. Phytopathology.

[15] Correa R.L., Silva T.F., Simoes-Araujo J.L., Barroso P.A., Vidal M.S., Vaslin M.F. (2005). Molecular characterization of a virus from the family *Luteoviridae* associated with cotton blue disease. Arch. Virol..

[16] Yang X., Wang Y., Guo W., Xie Y., Xie Q., Fan L., Zhou X. (2011). Characterization of small interfering RNAs derived from the geminivirus/betasatellite complex using deep sequencing. PLoS ONE.

[17] Zerbino D.R., Birney E. (2008). Velvet: Algorithms for de novo short read assembly using de Bruijn graphs. Genome Res..

[18] Tamura K., Dudley J., Nei M., Kumar S. (2007). MEGA4: Molecular Evolutionary Genetics Analysis (MEGA) software version 4.0. Mol. Biol. Evol..

[19] Muhire B.M., Varsani A., Martin D.P. (2014). SDT: A virus classification tool based on pairwise sequence alignment and identity calculation. PLoS ONE.

[20] Tabassum A., Roberts P.M., Bag S. (2020). Genome sequence of cotton leafroll dwarf virus infecting cotton in Georgia, USA. Microbiol. Resour. Announc..

[21] Ali A., Mokhtari S. (2020). Complete genome sequence of cotton leafroll dwarf virus isolated from cotton in Texas, USA. Microbiol. Resour. Announc..

[22] Avelar S., Ramos-Sobrinho R., Conner K., Nichols R.L., Lawrence K., Brown J.K. (2020). Characterization of the complete genome and P0 protein for a previously unreported genotype of cotton leafroll dwarf virus, an introduced polerovirus in the United States. Plant Dis..

[23] Distefano A.J., Bonacic Kresic I., Hopp H.E. (2010). The complete genome sequence of a virus associated with cotton blue disease, cotton leafroll dwarf virus, confirms that it is a new member of the genus *Polerovirus*. Arch. Virol..

[24] Mukherjee A.K., Mukherjee P.K., Kranthi S. (2016). Genetic similarity between cotton leafroll dwarf virus and chickpea stunt disease associated virus in India. Plant Pathol. J..

[25] Tabassum A., Bag S., Suassuna N.D., Conner K.N., Chee P., Kemerait R.C., Roberts P. (2021). Genome analysis of cotton leafroll dwarf virus reveals variability in the silencing suppressor protein, genotypes and genomic recombinants in the USA. PLoS ONE.

[26] Ll D., Andrew M.Q.K., Elliot L., Michael J.A., Carstens E.B. (2012). Family Luteoviridae. Virus Taxonomy: Ninth Report of the International Committee on Taxonomy of Viruses.

[27] SYLLER J. (2012). Facilitative and antagonistic interactions between plant viruses in mixed infections. Mol. Plant. Pathol..

[28] Savenkov E.I., Valkonen J.P. (2001). Potyviral helper-component proteinase expressed in transgenic plants enhances titers of Potato leaf roll virus but does not alleviate its phloem limitation. Virology.

[29] Barker H. (1987). Invasion of non-phloem tissue in *Nicotiana clevelandii* by potato leafroll luteovirus is enhanced in plants also infected with potato Y potyvirus. J. Gen. Virol..

[30] Ryabov E.V., Fraser G., Mayo M.A., Barker H., Taliansky M. (2001). Umbravirus gene expression helps potato leafroll virus to invade mesophyll tissues and to be transmitted mechanically between plants. Virology.

