# Supplementary material for: Coinfection of Cotton Plants with Watermelon Mosaic Virus and a Novel Polerovirus in China
Source: Viruses. 2021 Nov 3;13(11):2210. doi: 10.3390/v13112210 (PMC8618073; doi:10.3390/v13112210)
Supplement: Supplementary file 1 [file viruses-13-02210-s001.zip › viruses-1394551-supplementary.pdf]

viruses

MDPI

Coinfection of Cotton Plants with Watermelon Mosaic virus and a Novel Polerovirus in China

Table S1. Sequences of primers used for amplification of cotton leaf roll virus (CLRV) and watermelon mosaic virus (WMV).

Primer Name	Primer Sequences (5′–3′)
CLRV-1F	ATGTTGAATTTGATTATCTGC
CLRV-1R	TAGCAACTACATCTTCGGCGGT
CYV-2F	GCGAATCAGATGAGTACCACCG
CLRV-2R	CTCCATCTTCATCATCACATGT
CLRV-3F	CACATTGGCGGCGACTGCGATG
CLRV-3R	CACCGAAACCCCAGGGAGAGT
5′RACE CLRV	GATTACGCCAAGCTTCGCGGGTCTGTCTACCAGTGGCCGCTCG
3′RACE CLRV	GATTACGCCAAGCTTGGCTTGGAGGCCAGGGACAGGTCCTGG
WMV-1F	AATGGCAACAATTATGTTTGGA
WMV-1R	ATAAGGAGTATGTACGGATCTTC
WMV-2F	TGACAGTTGGGTATCATGT
WMV-2R	GTCGTTCATGAACTTCTTTCC
WMV-3F	TGACTGTACCAAAGTTGATATC
WMV-3R	TGGCATCCAGAACCCATCTCTC
WMV-4F	CAGAGTAGAAAGGAGAGATGGG
WMV-4R2	GCAACAAACATTACCGTACCTC
5′ RACE WMV	GATTACGCCAAGCTTGCGCTCAGGCCCGCGCACCTTTCTGTG
3′ RACE WMV	GATTACGCCAAGCTTGGCCGCAGCTCTCGCGGGAATTAACAGC
WMV-NS-RT	ggcagtatcgtgaattcgatgcCAGAGTAGAAAGGAGAGATGGG
WMV-NS-tag	GGCAGTATCGTGAATTCGATGC
WMV-NS-R	AACTGTCGAACTGTGAACCATC
qRT-CLRV-F	GTACAAGTGGGAAGACGAGAAA
qRT-CLRV-R	AAGCTGGGATCACGAAGTATG
qRT-WMV-F	GCTAAGACAGCTACGCAACTAC
qRT-WMV-R	CGGCATCACTCCTCTCATTATC
qRT-GbUBQ7-F	GACCTACACCAAGCCCAAGAAG
qRT-GbUBQ7-R	TGAGCCCACACTTACCACAATAGT
